# Navigating Disrupted Puberty: Development and Evaluation of a Mobile-Health Transition Passport for Klinefelter Syndrome

**DOI:** 10.3389/fendo.2022.909830

**Published:** 2022-06-24

**Authors:** Andrew A. Dwyer, Vanessa Héritier, Sofia Llahana, Lauren Edelman, Georgios E. Papadakis, Laurent Vaucher, Nelly Pitteloud, Michael Hauschild

**Affiliations:** ^1^William F. Connell School of Nursing, Boston College, Chestnut Hill, MA, United States; ^2^Endocrinology, Diabetes & Metabolism Service of the Department of Medicine, Lausanne University Hospital Centre Hospitalier Universitaire Vaudois (CHUV), Lausanne, Switzerland; ^3^Pediatric Endocrinology, Diabetes and Obesity Unit, Department of Women-Mother-Child, Lausanne University Hospital Centre Hospitalier Universitaire Vaudois (CHUV), Lausanne, Switzerland; ^4^School of Health and Psychological Sciences, City University of London, London, United Kingdom; ^5^Reproductive Medicine Unit, Department of Obstetrics and Gynecology, Lausanne University Hospital Centre Hospitalier Universitaire Vaudois Centre Hospitalier Universitaire Vaudois (CHUV), Lausanne, Switzerland

**Keywords:** adolescent, continuity of care, puberty, Klinefelter syndrome (KS), transition

## Abstract

Klinefelter syndrome (KS) is the most common aneuploidy in men and has long-term sequelae on health and wellbeing. KS is a chronic, lifelong condition and adolescents/young adults (AYAs) with KS face challenges in transitioning from pediatric to adult-oriented services. Discontinuity of care contributes to poor outcomes for health and wellbeing and transition programs for KS are lacking. We aimed to develop and test a mobile health tool (KS Transition Passport) to educate patients about KS, encourage self-management and support successful transition to adult-oriented care. First, we conducted a retrospective chart review and patient survey to examine KS transition at a university hospital. Second, we conducted a systematic scoping review of the literature on AYAs with KS. Last, we developed a mobile health transition passport and evaluated it with patient support groups. Participants evaluated the tool using the System Usability Scale and Patient Education Materials Assessment Tool (PEMAT). Chart review identified 21 AYAs diagnosed between 3.9-16.8 years-old (median 10.2 years). The survey revealed only 4/10 (40%) were on testosterone therapy and fewer (3/10, 30%) had regular medical care. The scoping review identified 21 relevant articles highlighting key aspects of care for AYAs with KS. An interprofessional team developed the mobile-health KS transition passport using an iterative process. Support group members (n=35) rated passport usability as ‘ok’ to ‘good’ (70 ± 20, median 73.5/100). Of PEMAT dimensions, 5/6 were deemed ‘high quality’ (86-90/100) and participants knew what to do with the information (actionability = 83/100). In conclusion, many patients with KS appear to have gaps in transition to adult-oriented care. Iterative development of a KS transition passport produced a mobile health tool that was usable, understandable and had high ratings for actionability.

## Introduction

Klinefelter syndrome (KS, 47, XXY) is the most common chromosomal disorder in males occurring in approximately 150 per 100,000 males (1:660) (1). Klinefelter, Reifenstein, and Albright first described KS in 1942 (2) and subsequent literature has expanded the variable phenotypic features and abnormalities observed in KS. The clinical constellation of small firm testes, primary (hypergonadotropic) hypogonadism, gynecomastia, infertility as well as a range of neuropsychiatric issues and learning disabilities are hallmark signs (3). The most common KS karyotype is 47 XXY and milder mosaic forms may be noted (4). As the degree of testicular insufficiency can be variable, clinical signs are subtle in many affected men limiting early and appropriate diagnosis. Indeed, a marked discrepancy was reported in a large Danish registry study when comparing KS prevalence in male fetus undergoing prenatal testing versus the postnatal diagnosis rate in the general population, suggesting that fewer than 25% of men with KS are diagnosed in a real-life setting (5).

KS can have wide-ranging effects on health and wellbeing (6, 7). The pituitary gonadal axis is disrupted (hypergonadotropic hypogonadism) and can manifest as disrupted puberty. There are striking metabolic disturbances with altered body composition and increased risk for insulin resistance and type 2 diabetes. Bone mineralization may be compromised and a spectrum of neurocognitive functional impairments as well as increased psychological and psychiatric morbidity are observed (6). Effective management requires a multidisciplinary approach that may include endocrinology, andrology/urology, neuropsychology, psychiatry, genetic counseling, nursing, social work, speech and language therapy, occupational therapy, and behavioral specialists (3). Only approximately 10% of cases are diagnosed during childhood (5). Patients diagnosed in childhood may exhibit more pronounced health and cognitive factors that challenge key developmental tasks. For adolescents, KS may affect developing autonomy, self-care skills, and care coordination during the transition from pediatric services to adult-oriented care. For young adults, gaps in care (e.g., “lost to follow-up”) can have significant negative sequelae on health and wellbeing (8). Structured approaches to transitional care for young adults with complex medical needs are needed to help patients develop self-management skills and support continuity of care (9). However, there are few published examples of structured transitional care for KS (10, 11). As such, there is limited information on best practices and evidence-based guidelines have yet to be established for KS transitional care.

To address the knowledge gap related to KS transition, we used a three-tiered approach. First, we conducted a retrospective chart review to examine the discontinuity in transitional care for patients with KS. Second, we conducted a systematic scoping review to evaluate the available evidence and recommendations for adolescents and young adults (AYAs) with KS during the transition to adult-oriented care. Third, we developed a novel mobile health (m-health) ‘transition passport’ to support and guide patients in self-management during transition and to ensure information transfer. Finally, we sought to validate the transition passport with KS patient-support groups by examining usability, understandability, and actionability of the transition passport.

## Methods

This study was conducted in accordance with the Declaration of Helsinki and was approved by local ethics committees (Lausanne University Hospital Ethics Committee and Boston College Institutional Review Board). All participants provided informed consent prior to study activities. The study was comprised of three parts. First, we conducted a chart review of the Lausanne University Children’s Hospital (Switzerland) and surveyed patients about their experiences during transition to adult-oriented care. The results of part I pointed to gaps in care and challenges during transition that spurred us to develop a mobile-health tool to support transition for AYAs with KS. The second part of the study involved a systematic scoping review examining the literature on transitional care for patients with KS. This was used to gather the best available evidence for creating the mobile-health tool. Last, we created a transition passport for patients with KS and validated it with patient support organizations.

### Chart Review and Patient Survey

To better understand the needs of patients with KS during transition we conducted a retrospective, descriptive follow-up study of AYAs with KS (>16 yrs.) seen in the pediatric endocrine service of the Lausanne University Children’s Hospital. Patients were identified by searching the hospital database of patients (2000-2014). Patients after 2014 were not included - as a structured transition program (Center for Endocrinology and Metabolism in Young Adults, CEMjA) was launched in 2014 (11). Individual chart review was performed to collect data on timing of diagnosis, medical management, and continuity of care (i.e. medical follow-up). Identified patients were mailed a previously validated questionnaire (12) that was adapted for KS. Briefly, the adapted 32-item instrument included 14 items on patient experiences with KS medical management (and current health), 5 general transition items, 6 KS-specific transition items, and 7 items on how transition could be improved (**Supplemental Material 1**).

### Systematic Scoping Review

To review the data and evidence base related to transitional care for AYAs with KS, we conducted a scoping review (AD, SL) according to the five-stage Arksey and O’Malley framework (13). Findings are reported according to the Preferred Reporting Items for Systematic Reviews and Meta-Analyses (PRISMA) (14). In brief, the scoping review involved five steps: 1) identifying the research question (“What impact does a structured transition to adult care have on continuity of care, health, and quality of life for young adults with Klinefelter syndrome?”), 2) identifying the relevant literature (systematic literature search using keywords related to KS and transition), 3) selecting the literature (independent investigators screened articles using Rayyan software (15), 4) charting the data (data was extracted from selected articles), and 5) collating, summarizing, and reporting results (evidence summarized in a table). **Supplemental Material 2** provides a detailed description of methods used for the scoping review of literature (1987-2017). Prior to manuscript preparation, the scoping review was repeated (2017-2021) to identify recently published studies (AD/LE) – reported in **Supplemental Material 3**.

### Transition Passport Development

An interprofessional team including pediatric/adult endocrinology, urology, and nursing engaged in an iterative process to develop content and design a mobile, electronic health (m-health) tool (i.e., KS transition passport) to support AYAs transition from pediatric to adult-oriented care. Broadly, the goal was to create an interactive tool (PDF) organized into easily navigable sections enabling patients to link/jump to preferred topics by clicking on icons - without having to read through the entire document (i.e., traditional printed materials). The KS transition passport serves as a psychoeducational tool enabling AYAs to learn about KS as well as a means to document and record information essential to their medical management. The interactive PDF format was chosen for reasons of simplicity as the format can be used with standard mobile electronic devices and independent of web connectivity and/or linkage to electronic health records. Language editing, word choice, layout, and design elements were modified and refined in an iterative development process. The final version (V.20) was translated from French to English. We assessed readability using algorithms that calculate the number of difficult words/sentences to provide an estimated age range and grade reading level. There is no accepted “gold standard” algorithm to assess readability so we utilized several validated instruments: Automated Readability Index, Linsear Write Formula, Flesch-Kincaid Grade Level, The Coleman-Liau Index, Flesch Reading Ease, and Gunning Fog Index and reported the calculated consensus reading level as previously reported (16).

### Evaluation of Transition Passport

To evaluate the transition passport, we partnered with patient organizations and used a web-based approach to reach geographically dispersed patients (17). Adult patients and parents/guardians able to read/write English were included for the evaluation of the transition passport. After providing electronic opt-in consent, participants were asked to review the transition passport and were informed that the purpose of the m-health tool was to support AYAs during transition to adult-oriented care. Participants viewed a pdf of the transition passport and completed a QualtricsTM survey to record sociodemographic information and several measures. Subjective health literacy was assessed as described by Chew and colleagues (18). This brief instrument has been shown to detect limited health literacy as assessed by lengthier validated instrument (Rapid Estimate of Adult Literacy in Medicine, AUROC=0.82) (19). Objective health literacy was measured using the Newest Vital Sign (NVS) (20). The NVS is a 6-item instrument that is widely used to identify individuals with limited health literacy and numeracy skills. Briefly, individuals are presented with a nutrition label and are required to identify and interpret basic text and perform simple mathematical computations. Scores identify adequate (score = 4-6), possibly limited (score = 2-3), or high likelihood (50%) chance of limited health literacy/numeracy (score = 0-1). The NVS correlates with the lengthier validated instrument (Test of Functional Health Literacy in Adults, AUROC=0.88) (20) and has good internal consistency (literacy: α=0.91, numeracy: α=0.78) (21).

To assess usability of the transition passport, participants completed the System Usability Scale (SUS) (22). Users respond to 10 questions (alternately worded positively and negatively) and record strength of agreement using a 5-point Likert-type scale. Scores are transformed into an overall score (0-100). The instrument is reliable (α=0.85) and is widely used to measure usability and satisfaction. Participants also completed the “gold standard” Patient Education Materials Assessment Tool (PEMAT). The PEMAT is a validated, 17-item instrument developed by the U.S. Department of Health & Human Services Agency for Health Research & Quality to evaluate print/audiovisual educational materials (23). After reviewing the materials, participants select agree/disagree/not-applicable to items assessing six domains relating to understandability (ability to process key messages - i.e. content, style, use of numbers, organization, design, and use of visual aids) and a domain on actionably (ability to identify steps one can take in response to presented information). Cumulative scores are expressed as a percentage (total score/possible total X 100). Psychometric evaluation demonstrates strong internal consistency, good reliability, and evidence of construct validity (23).

### Statistical Analyses

Data are reported using descriptive statistics. Student’s T-tests and Mann-Whitney rank sum tests were used (as appropriate) to compare SUS ratings between patients and parents/guardians. PEMAT scores ≥80% on a given parameter were deemed be ‘high-quality’ (16). Chi square and Fisher exact tests were used as appropriate to compare patients and parents/guardians for subjective/objective health literacy and PEMAT ratings. A *p* value <0.05 was considered statistically significant.

## Results

### Chart Review and Patient Survey

The retrospective search of the Lausanne University Hospital (CHUV) pediatric endocrine database identified 21 White non-Hispanic patients with KS (2000-2014). Age at initial consultation ranged from 3.9-16.8 years-old (median 10.2 yrs.). Of those responding to the survey, more than half of patients (9/14, 64%) received testosterone therapy in the pediatric setting. The duration of care in pediatrics ranged from 1-11 yrs. (mean 4.5 ± 3.5, median 4 yrs.). A survey was sent to patients and 10/14 (71%) completed the survey. Half of patients (5/10, 50%) reported being clinically followed in adult endocrinology and 3/10 (30%) reported that they did not seek regular medical care. Only 4/10 (40%) had ongoing testosterone treatment. We inquired about the preferred timing of transition and the majority of patients preferred transition between 16-19 yrs. (16-17 yrs.: n=4/10, 18-19 yrs.: 3/10). In terms of the transition process, results were divided. Half of patients (5/10) preferred to see the adult provider alone while the other half preferred to have parents included in medical visits.

### Systematic Scoping Review

To explore the survey findings suggesting gaps in care and challenges with adherence to treatment, we conducted a systematic scoping study of the available literature on AYAs with KS (1987-2017). From the initial review of 134 articles, 22 were included for full review and data extraction. The publications were from Australia, Belgium, France, Netherlands, Switzerland, and the United States. Data were extracted from articles on AYAs with KS including 11 review articles (8, 24–33), two systematic review/meta-analyses (34, 35), 6 cross-sectional/observational studies (36–41) and two interventional studies (42, 43). Most articles (13/21, 62%) were published between 2012-2017, pointing to a growing attention to the care of AYAs with KS. Indeed, following the evaluation of the KS transition passport (below) we repeated the structured literature search for publications 2017-2021 (**Supplemental Material 3**). Notably, there is no “gold standard” approach to treatment for AYAs with KS and evidence-based consensus guidelines are lacking (8, 31). From our initial scoping review, the most frequently addressed topics included psychological aspects of KS, fertility, and puberty. Considerations for bone, metabolic, autoimmune, and hematologic issues were less frequently discussed. Psychological/psychiatric aspects of KS were the most frequently examined (16/21, 76%) in identified articles (8, 24–32, 36, 37, 39–41, 43). A number of articles highlighted the psychosocial impact, health-related quality of life (HR-QoL), depressive symptoms, and adaptation to living with KS. Aspects of care relating to fertility were the second most frequently addressed in articles (13/21, 62%) (8, 28–30, 32–35, 38, 41–43). Infertility treatment (i.e., testicular sperm extraction, sperm retrieval rates, cryopreservation) was the dedicated topic of 6/21 (29%) of publications (33–35, 38, 42, 43). Of note, all (4/4) interventional studies and systematic reviews/meta-analyses focused on infertility treatment. Considerations relating to puberty (and testosterone replacement) were discussed in 9/21 (43%) (8, 25, 28–32, 39, 41), while metabolic (29, 31–33), bone health (8, 31, 32), and autoimmune disorders (8, 31) were less frequently noted. The available evidence/expert opinion of identified articles is charted in **Table 1**.

**Table 1:** Summary of findings from the systematic scoping review (n=21, 1987-2017).

ref #	year	design	pub.	fertil.	psych.	metab.	bone	Imm.	Summary of findings [country]
(24)	1989	review	–	–	YES	–	–	–	[USA] n=16 studies (only 1 specifically on AYAs), increased timidity, low confidence, late development of sexual interest, severe psychiatric illness is rare
(25)	2002	review	YES	–	YES	–	–		[USA] KS variable, most do not differ significantly from peers, intelligence is normal, problems may include low muscle tone, poor coordination, speech delays, low self-esteem, delayed sexual interest, initiation of TRT is important for AYAs
(26)	2004	review	–	–	YES	–	–	–	[USA] Only general information (not specific to KS), general ‘good clinical practices’ for psychological support during transition
(27)	2004	review	–	–	YES	–	–	–	[USA] Internalizing (anxiety, depression, withdrawal), social/emotional difficulties, inhibition/Bx issues, language-based learning difficulties, dyslexia, ADD/ADHD, learning disability, executive dysfunction.
(36)	2006	observational	–	–	YES	–	–	–	[Australia] n=32 (median: 24.5 yrs. [13–45]), 53% on TRT, 34% with normal T levels, 69% had psychosocial issues, 28% had impulse control issues, frequently lost to F/U with psychiatric services, TRT associated with improved mood, less depressive symptoms
(28)	2010	review	YES	YES	YES	–	–	–	[USA] General overview of TC and barriers, KS specific aspects include: TRT, infertility, T2DM, low BMD, breast/mediastinal cancers, and psychological support for learning difficulties and low self-esteem
(29)	2011	review	YES	YES	YES	YES	–	–	[USA] hypogonadism often appears in AYAs, cryptorchidism (5-69%), increased fat mass common, role of TRT on neuropsychological status is unclear
(37)	2011	observational	–	–	YES	–	–	–	[USA] n = 310 (40.7±14 yrs. [14-75]), 69% had significant depressive symptoms (CES-D), depressive symptoms were significantly associated with emotion-focused coping strategies/perceptions of stigmatization, perceived negative consequences of KS (IPQ), and infertility
(34)	2012	systematic review	–	YES	–	–	–	–	[USA] n=16 studies, overall 51% SRR (better rates with micro-TESE), no recommended age for SRR, positive SRR predictors = age <35yrs., near-normal T levels, response to hCG pre-op; negative predictors = low T at Dx
(38)	2012	observational	–	YES	–	–	–	–	[Belgium] n = 7 (13-16 yrs.), all biopsies showed IF, SPGA found in 1/7, authors propose testicular tissue preservation ideally be done before adolescence (yet no evidence on fertility outcomes).
(30)	2014	review	YES	YES	YES	–	–	–	[France] cryptorchidism increased in KS, testicular growth ends in mid-puberty, 15yrs. appears reasonable as lower limit for fertility preservation given psychosexual maturity, prior reports of increased psychiatric disease/mental retardation/criminality have not been confirmed by longitudinal studies
(8)	2014	review	YES	YES	YES	YES	YES	YES	[Belgium] multidisciplinary approach is important, high risk for lost to F/U, screen T in adolescence and initiate TRT as needed, SA can begin at 14yrs., screen AYAs for MetS/T2DM and associated diseases (autoimmune), begin DXA screening at end of puberty, increased VTE risk with KS, psychosocial support and focus on self-esteem during TC
(31)	2015	review	YES	YES	YES	YES	YES	YES	[Switzerland] Review of multiple hypogonadal states, importance of structured TC, puberty is normal in most KS, hypogonadism first evident in AYAs, increased risk for MetS, T2DM, decreased BMD, some autoimmune conditions, disrupted puberty can have psychological burden (victimization/bullying), provide anticipatory guidance/emotional support and openly discuss patient concerns during TC
(39)	2015	observational	YES	–	YES	–	–	–	[USA] n = 43 (8-18 yrs.), 70% had learning difficulties, 67% had speech & language problems, 63% had social interaction problems, 67% had impaired HR-QoL, 38% had low self-esteem, 26% had poor selfconcept, increased risk for depression, T levels not associated with psychosocial health measures
(40)	2015	observational	–	–	YES	–	–	–	[USA] n=310 (40.7 yrs. [14-75]), 76% had significant negative consequences of KS (IPQ), 69% had significant depressive symptoms (CES-D), 64% had high levels of adaption, 56% used emotion focused coping strategies, use of problem-focused coping strategies was the greatest predictor of adaptation, reframing cognitive appraisals may promote problem-focused coping and improve adaptation
(42)	2015	interventional	–	YES	–	–	–	–	[France] n=41 (15-22yrs: n=16, >16yrs: n=25), recommend TESE be discussed/offered (<35yrs.), overall SRR 50%, unclear evidence regarding timing of D/C TRT before TESE, 10 studies report SRR in AYAs (success: 0-70%), SRR higher for boys >15yrs., 8% with non-mosaic KS had sperm in the ejaculate
(43)	2016	interventional	–	YES	YES	–	–	–	[USA] n=28 (12-25yrs: n=15), 10/15 (66%) AYAs underwent micro-TESE, SRR rate 50%, no association between SRR and hormonal markers or TV, significantly more overall difficulties/symptoms (SDQ), 60% had an IEP, 40% had received MH services, 27% had ADD
(32)	2016	review	YES	YES	YES	YES	YES	–	[USA] most AYAs with KS enter puberty normally, TRT should be prescribed per ES guidelines, infertility is common in KS, SRR by TESE 54%, SRR optimal during peak Leydig cell function, decreased BMD
(33)	2016	review	–	YES	–	–	–	–	[Belgium/USA] pro/con debate, limited data available, SPGA stem cell/testicular tissue freezing/*in vitro* maturation strategies require further validation, testicular tissue from AYAs is controversial and should only be performed in a research framework, many ethical considerations
(35)	2016	systematic review	–	YES	–	–	–	–	[Netherlands] n=76 studies, KS often Dx in childhood due to Bx issues vs. infertility in adults, overall SRR rates are low for AYAs (n=10 studies, 0-70%), pre-pubertal TESE should not be offered (psychological impact), counselling should be provided in advance of SRR/TESE, 4 studies showed no spermatozoa in AYA ejaculate (10-25yrs: n=56). SRR higher for AYAs >15 yrs., only SPGA found were in children, 8% of non-mosaic KS have sperm in ejaculate, overall SRR in KS is 50%
(41)	2017	observational	YES	YES	YES	–	–	–	[USA] N = 310 (14-24yrs : n=31), Age and time of Dx were not predictive of psychological well-being, social impact similar in AYAs and adults, 21% said “worst” part of KS were small TV/gynecomastia/ height/low muscle mass due to victimization/bullying, 31% said infertility was the greatest challenge, 31% said psychological impact of KS Dx was the greatest challenge, 23% reported learning difficulties, 22% reported social problems, disclosing Dx to others was challenging but ultimately strengthened relationships and supported adaption to life with KS

headers Pub, puberty; Fertil, fertility; Psych, psychological/psychosocial; metab, metabolism; Imm, autoimmune disorders; table ADD/ADHD, attention deficit disorder/attention deficit hyperactivity disorder; AYAs, adolescents and young adults; BMD, bone mineral density; Bx, behavior; CES-D, Center for Epidemiologic Studies Depression; Dx, diagnosis; DXA, dual X-ray absorptiometry; ES, Endocrine Society; F/U, follow-up; hCG, human chorionic gonadotropin; HR-QoL, health-related quality of life; IEP, individualized educational program; IF, interstitial fibrosis; IPQ, Illness Perceptions Questionnaire; MetS, metabolic syndrome; MH, mental health; micro-TESE, microdissection testicular sperm extraction; SDQ, Strengths and Difficulties Questionnaire; SPGA, spermatogonia; SRR, sperm retrieval rate; T, testosterone; T2DM, type 2 diabetes mellitus; TC, transitional care; TESE, testicular sperm extraction; TRT, testosterone replacement therapy; TV, testicular volume; VTE, venous thromboembolism.

### Transition Passport Development

Spurred by the findings of the chart review and drawing on scoping review findings, an interprofessional team (endocrinology, urology, nursing) developed a KS transition passport to support effective transition to adult-oriented care. A series of face-to-face meetings charted the structure of the passport (an interactive PDF, **Supplemental Materials 4**, **5**) and topical material for the passport. Each section is matched with a representative icon for visual appeal and to decrease reading burden. Sections include informational content providing a brief overview of KS, chapters dedicated to specific developmental periods (at birth, infancy, childhood, puberty, young-adulthood, adulthood) and a summary of tests with recommended timing/frequency. Additional sections provide interactive components for patients to record information related to their care: ‘my follow up’ (dates for visits across specialty), ‘my treatments’ (type and timing), ‘my results’ (recent test results) and ‘my contacts’ (information for providers across disciplines and links to patient organizations). After multiple revisions, the final version (V.20) was translated from French to English and a single page ‘user guide’ was created to provide orientation to the KS transition passport (**Supplemental Material 4**). Assessing the readability of the final version (V.20) (**Supplemental Presentation 5**) revealed a range of readability scores (Automated Readability Index: 8.7 “8th- 9th grade reading level”, Linsear Write Formula: 8.9 “9th grade”, Flesch-Kincaid Grade Level: 9.7 “10th grade”, The Coleman-Liau Index: 11.0 “11th grade”, Flesch Reading Ease score: 51.4 “fairly difficult to read”, Gunning Fog: 12.8 “hard to read”). The automated consensus score of the algorithms yielded a rating of “fairly difficult to read” - consistent with a 10th grade (14-15 year-old reading level).

### Transition Passport Evaluation

**Figure 1:**
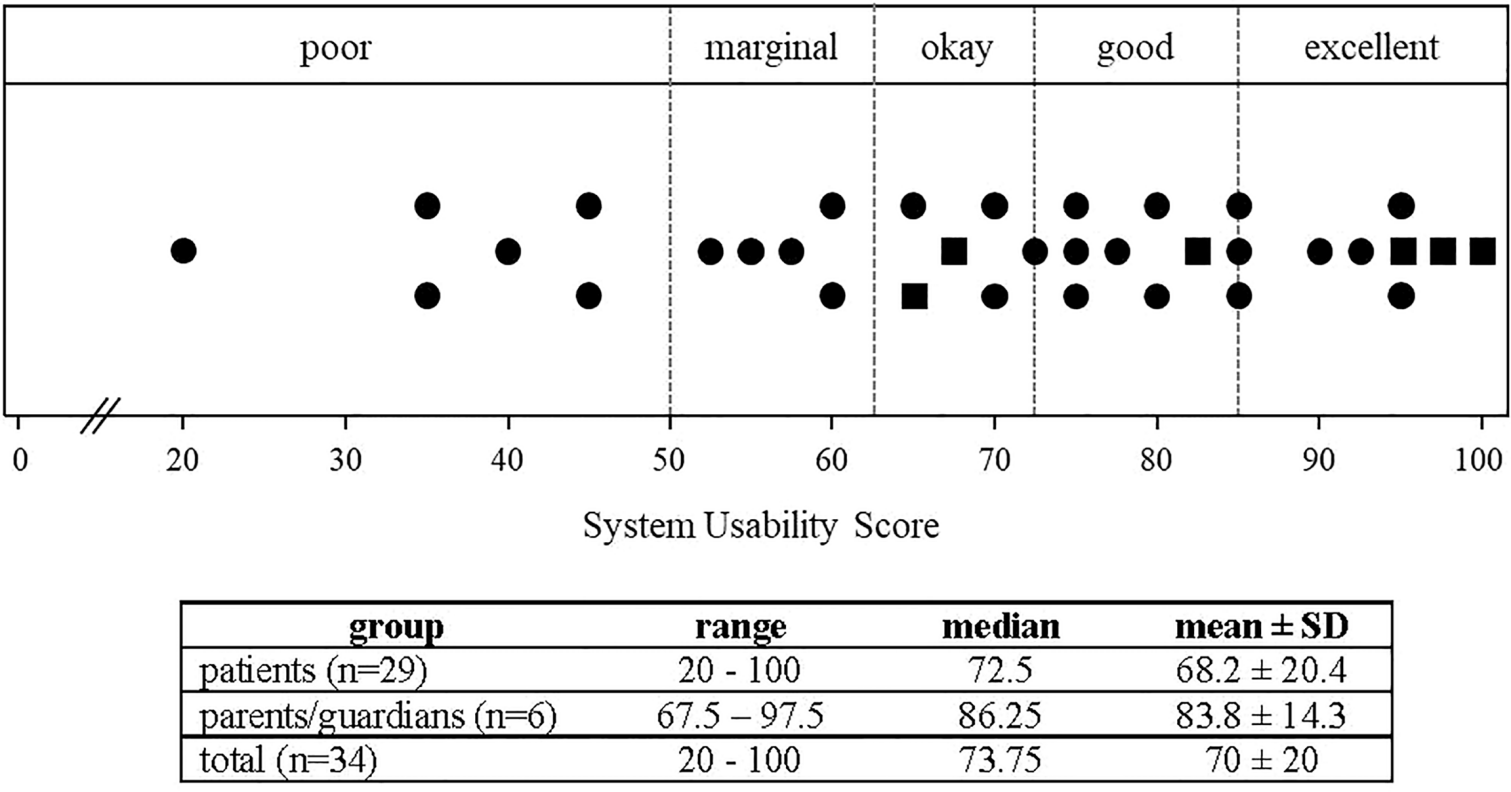
System Usability Scale (SUS) ratings of the KS transition Passport (n=34). Individual SUS ratings are shown for patients (circles) and parents/guardians (squares). Cutoffs are depicted by dotted lines. The table reports descriptive statistics of patient, parent/guardian and total ratings. Patient and parent/guardian SUS scores did not differ (p=0.094).

**Figure 2:**
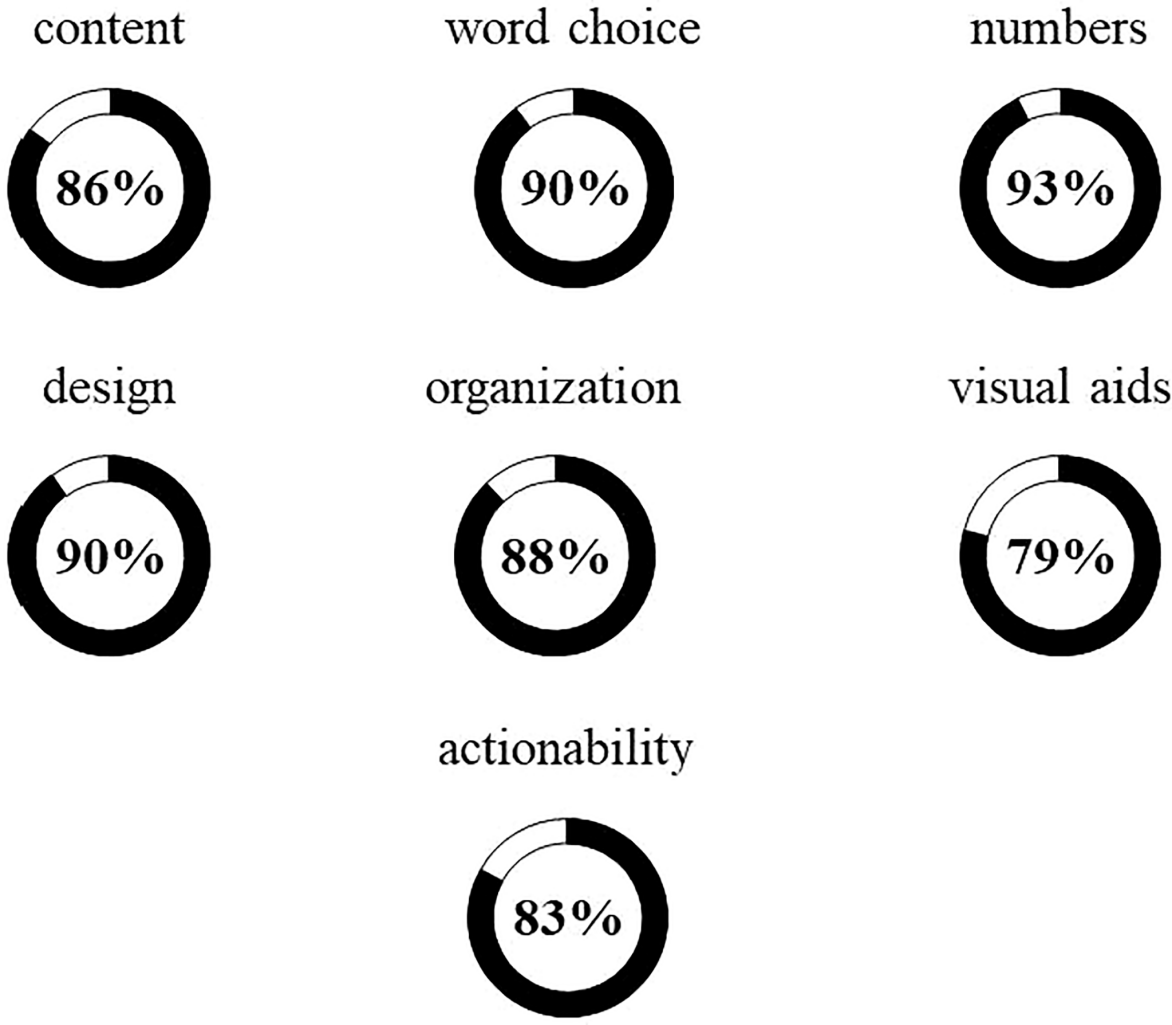
Patient Education Materials Assessment Tool (PEMAT) ratings of KS transition passport understandability and actionability (n=35). The PEMAT includes 17 items within seven domains. Six domains relate to understandability (top two rows) and the other is actionability. Approval ratings are depicted by circle areas in black and percent approval is noted in the center for each domain. Ratings ≥80% are considered 'high-quality'. Patient and parent/guardian PEMAT scores did not differ.

To evaluate the KS transition passport, we recruited subjects through several KS patient support organizations. In total, 35 participants completed the evaluation (**Table 2**). Overall, participants had relatively high levels of education with 27/35 (77%) having a college education or higher. Both subjective and objective health literacy were similarly high with >90% having adequate literacy/numeracy. No differences were observed between patients and parents/guardians in terms of objective health literacy/numeracy measures (NVS: p=0.37). Overall, participants rated usability (as assessed by the System Usability Scale) as ‘okay’ to ‘good’ (mean: 70 ± 20, median: 73.75) and ratings were similar between patients and parents/guardians (p=0.09) (**Figure 1**). We used the ‘gold standard’ Patient Education Materials Assessment Tool to assess understandability and actionability of the KS transition passport (**Figure 2**). Specific dimensions with scores >80 are considered ‘high-quality’. Five of six dimensions relating to understandability received scores >80 (range: 86-90/100). The ‘use of visual aids’ scored 79/100 and was the only PEMAT dimension below the *a priori* threshold of 80. Understanding what to do with the information (actionability) was rated 83/100. Patients with KS and parents/guardians exhibited similar PEMAT scores (*p*=0.9) – yet this observation should be interpreted with caution given the limited number of parent/guardian participants.

**Table 2:** Demographic characteristics of participants evaluating the KS transition passport (n=35).

Characteristic	n (%)
**Participants**	
patients	29 (83%)
age (yrs): mean±SD (median, range)	47.9±17.7 (50, 21-77)
age at diagnosis*: mean±SD (median, range)	28.0±14.5 (29, 1-61)
parents/guardians	6 (17%)
age (yrs): mean±SD (median, range)	58.3±16.8 (64, 20-77)
**education**	
less than college (high school/vocational) college post-graduate	8 (23%) 16 (46%) 11 (31%)
**subjective health literacy^†^**	
adequate inadequate	32 (91%) 3 (9%)
**objective health literacy – NVS** (n=25)	
adequate moderate low	23 (92%) 2 (8%) 0

*three patients diagnosed prenatally and were not included in the calculation.

^†^subjective health literacy (18). NVS: Newest Vital Sign (20).

## Discussion

This three-part study provides several important insights into the management of adolescents and young adults (AYAs) with KS. First, our retrospective chart review indicated that a significant portion of young men with KS struggled with transitioning from pediatric to adult-oriented care resulting in gaps in treatment and care. Second, the systematic review of the literature and scoping study highlighted a lack of consensus regarding transitional care of AYAs with KS. The paucity of high-quality data limits the development of evidence-based guidelines for practice. We developed a KS transition passport using an iterative process with an interprofessional team incorporating findings form the scoping review. Evaluating the KS transition passport with patient groups showed that the passport is usable, understandable, and actionable – and thus, may represent an important avenue for extending the reach of care for AYAs with KS.

Given the broad phenotypic spectrum of KS, some AYAs with KS may not differ significantly from their peers. Overt KS phenotype is often evident only after puberty or when fertility is pursued (25, 44). Subtle clinical presentation or mosaicism (4) may contribute to the estimated 75% of men with KS who remain undiagnosed (5). It is plausible to consider that earlier KS diagnosis is likely in more severely affected individuals. The present data support the notion that there are often gaps in transition. Such discontinuity of care can affect health outcomes and quality of life and may contribute to avoidable healthcare costs. A 2016 Cochrane review on transition of care for AYAs with special health needs concluded that effective transitional care has several key benefits including enhanced self-management as well as improved patient outcomes, satisfaction, and adherence to treatment (45). For AYAs with KS, gaps in care can have detrimental effects on sexual, bone, and metabolic health and can contribute to impaired health-related quality of life (8, 31). There is growing attention to the importance of transitional care for AYAs with KS as demonstrated by our initial scoping review in which 13/21 (62%) were published between 2012-2017. Further, the updated scoping review following evaluation of the KS transition passport identified 23 articles (2017-2021) supporting growing focus on AYAs and transitional care in recent years.

To support AYAs during transition (i.e., 16-25 yrs.), an interprofessional team developed the “KS transition passport”. The m-health tool was intended to serve as a psychoeducational support for therapeutic education to help patients better understand KS, as well as a resource promoting patient activation (i.e., empowerment) enabling patients to record vital health information during the transfer from pediatric to adult-oriented care. The passport can equally be used as a standardized information transfer tool between health professionals. The idea of using a “passport” to support AYAs during transition is not new. Indeed, the Lausanne University Children’s Hospital has developed and uses a transition passport for type 1 diabetes and Turner Syndrome. Other groups have published findings on similar passports for AYAs with diabetes (46), inflammatory bowel disease (47), congenital heart defects (48, 49), and osteogenesis imperfecta (50). While the KS Transition Passport is not an intervention to specifically improve physical and intellectual development, we envisioned it as a psycho-educational tool to enhance patient awareness and understanding of their condition and to support patient activation (i.e., PEMAT actionability domain). Notably, such patient activation is critical for empowering individuals for self-management of a chronic health condition and helps contribute to continuity of care as well as and improved health outcomes (51). We evaluated the KS transition passport with KS patient support organizations and found the usability of the eHealth tool was ‘good’. However, 6/34 (17.6%) respondents rated usability as ‘poor’. Half of those giving a ‘poor’ rating reported their highest level of education as vocational training – yet 6/6 had adequate subjective health literacy and 5/6 had adequate objective health literacy scores. Thus, a ‘poor’ usability rating does not appear to be linked to health literacy and additional usability enhancements could be made. In terms of the PEMAT, 5/6 dimensions assessing understandability and actionability (i.e., knowing what to do with the information) were considered ‘high-quality’ (i.e., >80%) suggesting high acceptability ratings. Similar to other studies evaluating transition passports, our findings provide initial support for the KS transition passport as a promising complement to structured transitional care for AYAs with KS.

Relative strengths of the study include the sequential process (i.e., retrospective chart review, systematic scoping review, iterative tool development and evaluation using ‘gold standard’ measures). The study has a number of limitations including the relatively small sample size for the retrospective chart review/survey (n=21) and the evaluation with KS patient organizations (n=35). We did not collect data on self-identified race/ethnicity. As such, the findings should not be considered to represent the perspectives of individuals with Klinefelter syndrome from diverse ancestry. Indeed, a recent review has identified noted paucity of data on the experiences patients with Klinefelter syndrome from and Black, Indigenous, People of Color (BIPOC) communities (52). Similarly, we did not capture whether or not individuals were treated with testosterone in the pediatric setting and cannot make any inference on the potential role of treatment on the evaluation of passport. It is worthwhile to note that the participants had high levels or health literacy/numeracy and may not be representative of all patients and parent/guardians. Patient-facing materials are recommended to be written at the 6-8th grade reading level (i.e., 11-13 yrs.). Despite the iterative process guided by principles of health literacy 51, we were only able to achieve a 10th grade (14-15 year-old) reading level. This is likely due to the fact that several complex, multisyllabic words needed to be stated and defined (e.g., chromosome, Klinefelter, testosterone, azoospermia, metabolic, osteoporosis). However, as we considered transition to encompass 16-25 years-old, the 14-15 year-old reading level of the passport may not overly be problematic. We did not observe any differences in PEMAT scores between patients and parents/guardians - yet this finding should be interpreted with caution given the limited number of participants. The mean age of diagnosis of KS patients evaluating the transition passport was 28 years-old and these men may have milder KS phenotypes.

The iterative development process involved an interprofessional team yet did not involve patient/parent stakeholders. This is relevant as involving patient stakeholders in a “design-thinking” (i.e., user-centered design) process can improve understandability, actionability and acceptability of co-created patient-facing materials 16. Almost a third (11/35, 31%) of patient/parents provided free-text comments that were generally positive and expressed appreciation for the work. However, a number of comments also noted a desire for greater detail regarding mosaicism, thrombosis risk, complexity of behavioral issues, how to find a specialist/ask for referrals, and starting an occupation. Indeed, comments from patients/parents requesting information and greater detail on learning difficulties, academic supports, disability/function, and psychiatric diagnoses may reflect that behavioral health specialists (e.g., psychologists/psychiatrists, professionals in special education) were not involved in the iterative ‘design thinking’ process. In hindsight, inclusion of such perspectives could have strengthened the development of the m-health tool. Notably, patient-facing material need to be regularly reviewed and updated to ensure that the information accurately reflects the current state of the science and standards of care as well as unmet patient needs. Future work should include systematic follow up of AYAs following transition to adult-oriented care. Additionally, establishing rigorous criteria to identify uniform groups of patients with Klinefelter syndrome (i.e., clinical and molecular features) could support more targeted and rigorous clinical and fundamental research on Klinefelter syndrome.

In conclusion, many patients with KS appear to have gaps in transition to adult-oriented care. Iterative development of a KS transition passport produced a tool that was usable, understandable, and had high ratings for actionability. Such eHealth tools can support a comprehensive, structured approach to transitional care for AYAs with KS. Future work may examine the effect of the KS transition passport on knowledge, patient activation, adherence to treatment, and continuity of care.

## Data Availability Statement

The raw data supporting the conclusions of this article will be made available by the authors, without undue reservation.

## Ethics Statement

The studies involving human participants were reviewed and approved by Lausanne University Hospital Ethics Committee and Boston College Institutional Review Board. The patients/participants provided their written informed consent to participate in this study.

## Author Contributions

AD made substantial contributions to the study design, acquired and analysed the literature for the scoping review, contributed to the development of the transition passport, collected and analysed the data for the evaluation of the transition passport and drafted the manuscript. VH made substantial contributions to collecting and analysing the chart review and patient survey data, contributed to the development of the transition passport, provided critical feedback on the manuscript and approved the final manuscript. SL made substantial contributions acquiring and analysing the literature for the scoping review, provided critical feedback on the manuscript and approved the final manuscript. LE made substantial contributions to collecting and analysing the data for the evaluation of the transition passport, provided critical feedback on the manuscript and approved the final manuscript. GP, LV, and NP made substantial contributions to developing the transition passport, provided critical feedback on the manuscript and approved the final manuscript. MH made substantial contributions to the study design, led the development of the transition passport, provided critical feedback on the manuscript and approved the final manuscript. All authors contributed to the article and approved the submitted version.

## Funding

This research did not receive any specific grant from any funding agency in the public, commercial or not-for-profit sector. Open access funding provided by University of Lausanne.

## Conflict of Interest

The authors declare that the research was conducted in the absence of any commercial or financial relationships that could be construed as a potential conflict of interest.

## Publisher’s Note

All claims expressed in this article are solely those of the authors and do not necessarily represent those of their affiliated organizations, or those of the publisher, the editors and the reviewers. Any product that may be evaluated in this article, or claim that may be made by its manufacturer, is not guaranteed or endorsed by the publisher.

## Supplementary Material

Supplemental Material 1 Patient survey.

Supplemental Material 2 Scoping Review methods and PRISMA diagram (1987-2017).

Supplemental Material 3 Scoping Review (2017-2021) references and PRISMA diagram.

Supplemental Material 4 Passport instructions.

Supplemental Material 5 Klinefelter syndrome transition passport.
