# Erratum: Navigating disrupted puberty: Development and evaluation of a mobile-health transition passport for Klinefelter syndrome

**DOI:** 10.3389/fendo.2023.1182233

**Published:** 2023-03-15

**Authors:** Frontiers Production Office

**Affiliations:** Frontiers Media SA, Lausanne, Switzerland

**Keywords:** adolescent, continuity of care, puberty, Klinefelter syndrome (KS), transition

An omission to the funding section of the original article was made in error. The following sentence has been added: “Open access funding provided by University of Lausanne”.

The original version of this article has been updated.

