# Supplementary material for: Navigating Disrupted Puberty: Development and Evaluation of a Mobile-Health Transition Passport for Klinefelter Syndrome
Source: Front Endocrinol (Lausanne). 2022 Jun 24;13:909830. doi: 10.3389/fendo.2022.909830 (PMC9264386; doi:10.3389/fendo.2022.909830)
Supplement: Supplemental Material 1 — Patient survey. [file Presentation_1.pdf]

Supplementary Material 1.
Patient questionnaire from chart review (translated from French)

Please answer the following questions regarding your health

1. At what age were you diagnosed with Klinefelter Syndrome (KS)?

   ☐ I don't know ☐ at birth ☐ at age ______ years

2. How long have you been aware of the KS diagnosis?

   ☐ _______ years

3. In general, you rate your health as:

   ☐ excellent ☐ very good ☐ good ☐ average ☐ poor

4. In the past 12 months, how often have you missed school or work because of a problem related to your KS?

   ☐ never

   ☐ almost never

   ☐ approximately once per month

   ☐ approximately once per week

   ☐ several times per week

5. How much do you think that your KS affects your daily life?

   ☐ not at all ☐ a little bit ☐ moderately ☐ quite a bit ☐ a lot

6. How many times per year do you see your doctor (e.g. general practitioner, primary care provider, pediatrician) because of your KS?

   ☐ never ☐ 1-2 times ☐ 3-5 times ☐ More than 5 times ☐ I don't have a doctor

7. How many times per year have you consulted your KS specialist for children?

   ☐ never ☐ 1-2 times ☐ 3-5 times ☐ More than 5 times ☐ I don't have a specialist

8. How would you describe your relationship with your pediatric KS specialist?

9. Are you followed by a KS specialist for adults?

☐ yes ☐ no

10. If yes, which medical specialty (select all that apply)

☐ endocrinologist ☐ Fertility specialist ☐ Urologist ☐ Family Doctor

11. Are you on regular medications?

☐ no ☐ yes, daily (since ______ years-old) ☐ yes, monthly (since ______ years-old)

12. How many times a year do you see the specialist for your KS?

☐ never ☐ 1-2 times ☐ 3-5 times ☐ More than 5 times

13. Where do you consult your KS specialist for adults?

☐ at the hospital ☐ at his/her private practice

14. Before transitioning to an adult specialist, did you usually have the opportunity to talk to your doctor alone without your parents?

☐ always ☐ most of the time ☐ several times ☐ rarely ☐ never

☐ My parents did not accompany me to consultations

15. When consulting the KS specialist (or team), who is the most important person for you?

☐ doctor

☐ nurse

☐ social worker

☐ physical therapist

☐ other: ____________________

□ not applicable - I see the specialist alone in his/her private practice

16. If you are not followed by a KS specialist for adults, has your doctor spoken to you about a possible transition to adult care?

□ yes □ no

17. Why did you choose not to be followed by an adult specialist?

18. Before you started being followed by an adult KS specialist, at what age did your pediatric specialist first tell you about your transition to an adult specialist?

□ he/she never told me about transition □ at age: __________

19. How old were you when you transitioned to an adult specialist?

□ at age: __________

20. What options did your pediatric KS specialist give you about the first meeting with the adult specialist? (please select all that apply)

□ meet the adult specialist with the pediatric specialist in the adult clinic

□ meet the specialist for adults with the pediatric specialist at the children's hospital

□ meet with the adult specialist alone

□ I had to search for an adult specialist on my own

□ no options were given

21. How much did you feel supported during the transition to an adult KS specialist?

□ a lot □ quite a bit □ moderately □ a little bit □ not at all

22. In your opinion, where is the best place to meet an adult KS specialist for the first time?

□ at the children's hospital

□ at the adult hospital or the private clinic

□ I do not have a preference

23. In your opinion, what is the preferred way to meet an adult KS specialist for the first time?

□ meet the adult specialist in his/her hospital/practice with the pediatric specialist

□ meet the adult specialist in the children's hospital with the pediatric specialist

□ meet the adult specialist alone (without the pediatric specialist)

□ I do not have a preference

24. In your opinion, at what age should teenagers leave their pediatric specialist?

□ at or before the age of 13

□ at the age of 14-15

□ at the age of 16-17

□ at the age of 18-19

□ after the age of 20

25. In your opinion, what is the most important part of determining if a teenager is ready to transition to an adult KS specialist?

□ age

□ feeling "too old" to continue seeing a pediatric specialist

□ severity of KS

□ quality of the relationship between the teenager and the pediatric specialist

□ when parents think their child is ready

□ Other: ____

26. In your opinion, who should decide whether a teenager is ready to leave the pediatric specialist and start seeing an adult specialist?

□ patient only

□ patient and his parents

□ patient and the doctor

☐ doctor only

☐ parents only

☐ parents and the doctor

☐ patient, his parents, and the doctor

27. According to your experience, what are the obstacles to the transition of care in KS?

28. Who do you think should be involved in guiding you and keeping you informed when you change doctors? (select all that apply).

☐ pediatric KS specialist

☐ adult KS specialist

☐ nurse

☐ social worker

☐ physical therapist

☐ family doctor / general practitioner

☐ family

☐ Other: ____

29. What was your parent's role during this transition process?

30. Did you feel neglected during the transition to the adult KS specialist?

☐ not at all ☐ a little bit ☐ moderately ☐ quite a bit ☐ a lot

31. What could have been helpful during your transition to an adult KS specialist?

32. Have you spoken with your general practitioner about changing from a pediatric to an adult KS specialist?

☐ not at all ☐ a little bit ☐ moderately ☐ quite a bit ☐ a lot

33. Can we contact your family doctor/general practitioner?

☐ yes ☐ no
