# Supplementary material for: Navigating Disrupted Puberty: Development and Evaluation of a Mobile-Health Transition Passport for Klinefelter Syndrome
Source: Front Endocrinol (Lausanne). 2022 Jun 24;13:909830. doi: 10.3389/fendo.2022.909830 (PMC9264386; doi:10.3389/fendo.2022.909830)

Supplementary Material 2.
Scoping Review methods and PRISMA diagram (1987-2017)

A. Supplemental Methods:

The systematic scoping review followed the five-stage Arksey and O'Malley framework (1). The process for each of the five steps are described:

1. *Identifying the research question*:

To examine what aspects of health and wellbeing have been studied in young men with KS as well as the recommended practices for transition we used the following question to guide the literature search: "What impact does a structured transition to adult care have on continuity of care, health and quality of life for young adults with Klinefelter syndrome ?".

2. *Identifying relevant literature*:

The literature search was conducted in EMBASE, MEDLINE Ovid, PubMed, Cochrane Library, PsycINFO and CINAHL databases. A systematic, structured search was conducted using identified key words and articles were screened by two independent reviewers (AD, SL) using Rayyan software (2). Articles published up to February 2017 were retrieved. The key words and MeSH terms used for the search in the respective databases are identified below:

EMBASE: ('Klinefelter syndrome'/exp OR (klinefelter* OR (XXXXY NEXT/1 (syndrome* OR trisom* OR constitution*)) OR (XXXY NEXT/1 (syndrome* OR trisom* OR constitution*)) OR (XXY NEXT/1 (syndrome* OR trisom* OR constitution*))):ab,ti) AND ('young adult'/exp OR 'adolescent'/exp OR 'adolescence'/exp OR 'adolescent health'/exp OR ((Young NEXT/1 Adult*) OR (Prime NEXT/1 Adult*) OR Adolescen* OR Teen*):ab,ti) AND ('child health care'/de OR 'case management'/exp OR 'disease management'/exp OR 'clinical pathway'/exp OR ("Adolescent Health Service" OR "Adolescent Health Services" OR Transition* OR ((Continu* OR coordination) NEAR/3 care) OR ((Disease OR case OR disorder OR illness) NEAR/3 management) OR ((Critical OR clinical) NEXT/3 path*) OR (Patient NEXT/1 navigation*) OR ((child* OR pediatr* OR adolescen* OR teen*) NEAR/3 adult*) OR ((Transfer* OR service* OR care) NEAR/3 adult*)):ab,ti)

Medline Ovid: (exp "Klinefelter Syndrome"/ OR (klinefelter* OR (XXXXY ADJ (syndrome* OR trisom* OR constitution*)) OR (XXXY ADJ (syndrome* OR trisom* OR constitution*)) OR (XXY ADJ (syndrome* OR trisom* OR constitution*))).ab,ti.) AND ("Young Adult"/ OR "Adolescent"/ OR "Adolescent Health"/ OR ((Young ADJ Adult*) OR (Prime ADJ Adult*) OR Adolescen* OR Teen*).ab,ti.) AND ("Adolescent Health Services"/ OR exp "Continuity of Patient Care"/ OR "Disease Management"/ OR "Case Management"/ OR "Critical Pathways"/ OR "Patient Navigation"/ OR ("Adolescent Health Service" OR "Adolescent Health Services" OR Transition* OR ((Continu* OR coordination) ADJ3 care) OR ((Disease OR case OR disorder OR illness) ADJ3 management) OR ((Critical OR clinical) ADJ3 path*) OR (Patient ADJ navigation*) OR ((child* OR pediatr* OR adolescen* OR teen*) ADJ3 adult*) OR ((Transfer* OR service* OR care) ADJ3 adult*)).ab,ti.)

PubMed: (Klinefelter*[tiab] OR XXXXY Syndrome*[tiab] OR XXXY Syndrome*[tiab] OR XXY Syndrome*[tiab]) AND (Young Adult*[tiab] OR Prime Adult*[tiab] OR Adolescen*[tiab] OR Teen*[tiab]) AND (transition*[tiab] OR "Continuity of care"[tiab] OR Coordination of care*[tiab] OR "Continuity of Patient Care"[tiab] OR "Continuum of care"[tiab] OR Disease management*[tiab] OR Case Manag*[tiab] OR Critical Path*[tiab] OR Clinical Path*[tiab] OR Patient Navigation*[tiab] OR Disorder management*[tiab] OR Illness management*[tiab] OR Management of disease*[tiab]) NOT medline[sb]

Cochrane library : (klinefelter* OR (XXXXY NEXT/1 (syndrome* OR trisom* OR constitution*)) OR (XXXY NEXT/1 (syndrome* OR trisom* OR constitution*)) OR (XXY NEXT/1 (syndrome* OR trisom* OR constitution*))):ab,ti AND ((Young NEXT/1 Adult*) OR (Prime NEXT/1 Adult*) OR Adolescen* OR Teen*):ab,ti AND ("Adolescent Health Service" OR "Adolescent Health Services" OR Transition* OR ((Continu* OR coordination) NEAR/3 care) OR ((Disease OR case OR disorder OR illness) NEAR/3 management) OR ((Critical OR clinical) NEXT/1 path*) OR (Patient NEXT/1 navigation*) OR ((child* OR pediatr* OR adolescen* OR teen*) NEAR/3 adult*) OR ((Transfer* OR service* OR care) NEAR/3 adult*)):ab,ti

PsycINFO: [population limit : adolescent OR young adult] (klinefelters syndrome/ OR (klinefelter* OR (XXXXY ADJ (syndrome* OR trisom* OR constitution*)) OR (XXXY ADJ (syndrome* OR trisom* OR constitution*)) OR (XXY ADJ (syndrome* OR trisom* OR constitution*))).ab,ti.) AND ("continuum of care"/ OR disease management/ OR case management/ OR ("Adolescent Health Service" OR "Adolescent Health Services" OR Transition* OR ((Continu* OR coordination) ADJ3 care) OR ((Disease OR case OR disorder OR illness) ADJ3 management) OR ((Critical OR clinical) ADJ3 path*) OR (Patient ADJ navigation*) OR ((child* OR pediatr* OR adolescen* OR teen*) ADJ3 adult*) OR ((Transfer* OR service* OR care) ADJ3 adult*)).ab,ti.)

CINAHL: ((MH "Klinefelter's Syndrome") OR TI (klinefelter* OR (XXXXY W1 (syndrome* OR trisom* OR constitution*)) OR (XXXY W1 (syndrome* OR trisom* OR constitution*)) OR (XXY W1 (syndrome* OR trisom* OR constitution*))) OR AB (klinefelter* OR (XXXXY W1 (syndrome* OR trisom* OR constitution*)) OR (XXXY W1 (syndrome* OR trisom* OR constitution*)) OR (XXY W1 (syndrome* OR trisom* OR constitution*)))) AND ((MH "Young Adult") OR (MH "Adolescence+") OR (MH "Adolescent Health") OR TI ((Young W1 Adult*) OR (Prime W1 Adult*) OR Adolescen* OR Teen*) OR AB ((Young W1 Adult*) OR (Prime W1 Adult*) OR Adolescen* OR Teen*)) AND ((MH "Adolescent Health Services") OR (MH "Continuity of Patient Care") OR (MH "Transitional Care") OR (MH "Disease Management") OR (MH "Child Health Services") OR (MH "Case Management") OR (MH "Patient Navigation") OR (MH "Critical Path") OR TI ("Adolescent Health Service" OR "Adolescent Health Services" OR Transition* OR ((Continu* OR coordination) N3 care) OR ((Disease OR case OR disorder OR illness) N3 management) OR ((Critical OR clinical) W3 path*) OR (Patient W1 navigation*) OR ((child* OR pediatr* OR adolescen* OR teen*) N3 adult*) OR ((Transfer* OR service* OR care) N3 adult*)) OR AB ("Adolescent Health Service" OR "Adolescent Health Services" OR Transition* OR ((Continu* OR coordination) N3 care) OR ((Disease OR case OR disorder OR illness) N3 management) OR ((Critical OR clinical) W3 path*) OR (Patient W1 navigation*) OR ((child* OR pediatr* OR adolescen* OR teen*) N3 adult*) OR ((Transfer* OR service* OR care) N3 adult*)))

Database	references retrieved	references (no duplicates)
Medline Ovid SP	43	41
PubMed	0*	0*
Embase	113	84
PsycINFO	14	8
CINAHL	4	1
Cochrane Library	4	0
Total	178	134

* 0 denotes that all retrieved PubMed articles were identified in Medline Ovid

3. *Selecting the literature (inclusion-exclusion criteria)*

Articles included in this scoping review met specified inclusion criteria. All considered articles were published in English and related to adolescents/young adults with KS. Publications not specific to KS and foreign language papers were excluded. Case reports, meeting abstracts, drug studies, animal studies and reports of genetic results only were excluded from the analysis. The search strategy identified a total of 178 references. After removing duplicates, a total of 134 articles underwent title screening by two independent reviewers (AD, SL) using Rayyan software (2). Discussion to consensus was needed to determine relevance for a subset of 39 articles. In total, 29 relevant abstracts were reviewed yielding 22 articles for full text review and data extraction.

4. *Charting the Data*

Two reviewers (AD, SL) extracted data using a structured, standardized form capturing article title, authors, year, country, article type/study design, sample size, and outcomes and data related to six domains: puberty, fertility, psychological aspects, metabolic health, bone health, and autoimmune diseases. The independent investigators each read and extracted data from 11 articles each. Subsequently, the articles were swapped and reviewed by the opposite reviewer to assure data extraction accuracy.

5. *Collating, summarizing, and reporting the literature*

For each domain, we summarized findings in a table to provide context for the relevant weight and importance each topic has received in the literature as well as to summarize the available evidence and professional opinion on these topics related to KS (see **Table 1** in the manuscript). The search results are reported according to the Preferred Reporting Items for Systematic Reviews and Meta-Analyses (PRISMA)(3) – see PRISMA diagram below.

References

1. Levac D, Colquhoun H, O'Brien KK. Scoping studies: advancing the methodology. Implement Sci. 2010;5:69.
2. Ouzzani M, Hammady H, Fedorowicz Z, Elmagarmid A. Rayyan-a web and mobile app for systematic reviews. Syst Rev. 2016;5(1):210.
3. Moher D, Shamseer L, Clarke M, Ghersi D, Liberati A, Petticrew M, et al. Preferred reporting items for systematic review and meta-analysis protocols (PRISMA-P) 2015 statement. Syst Rev. 2015;4:1.

B. PRISMA flow diagram for literature search (1987-2017)

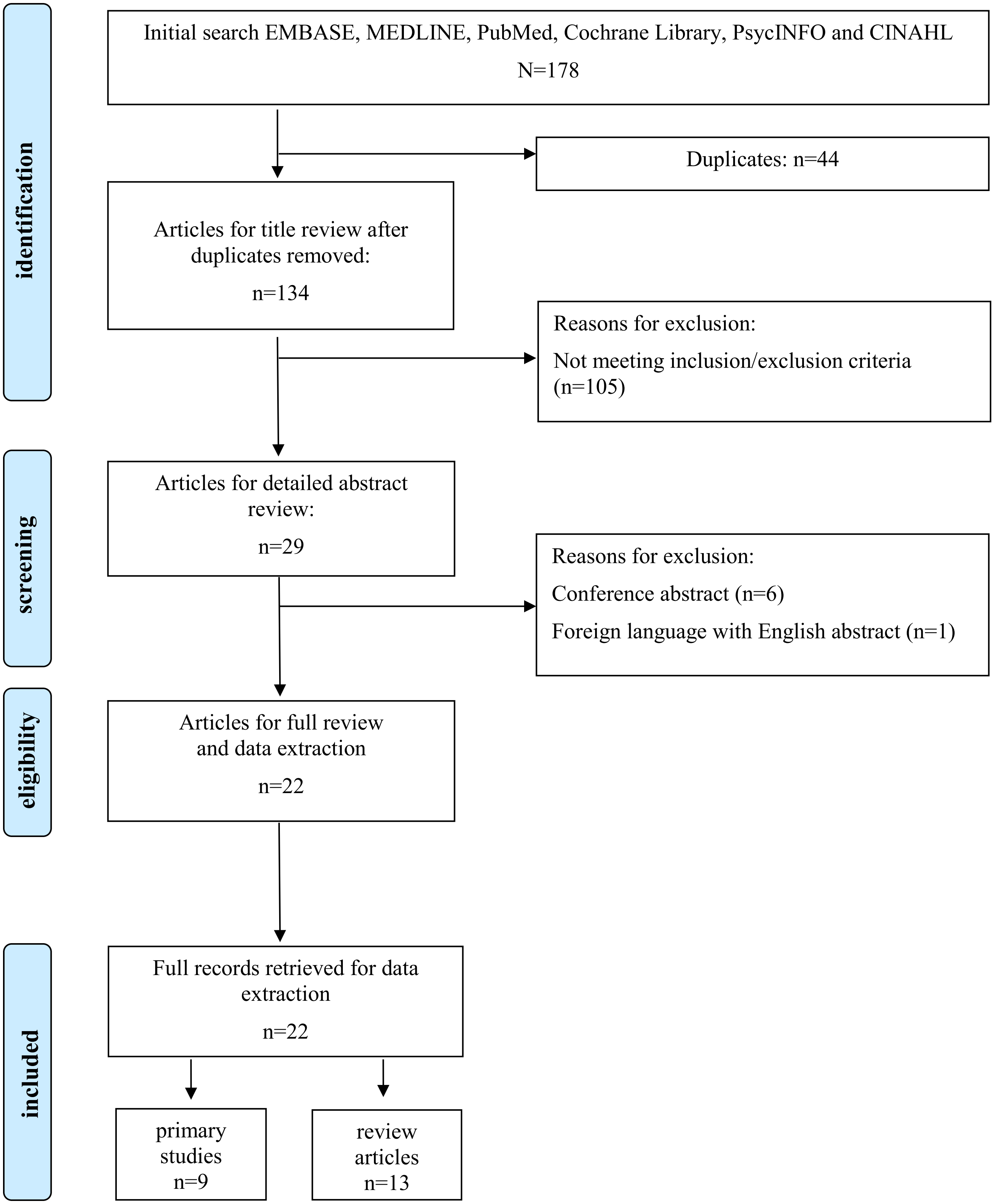

Supplement: Supplemental Material 2 — Scoping Review methods and PRISMA diagram (1987-2017). [file Presentation_2.pdf]
