# Supplementary material for: Navigating Disrupted Puberty: Development and Evaluation of a Mobile-Health Transition Passport for Klinefelter Syndrome
Source: Front Endocrinol (Lausanne). 2022 Jun 24;13:909830. doi: 10.3389/fendo.2022.909830 (PMC9264386; doi:10.3389/fendo.2022.909830)
Supplement: Supplemental Material 3 — Scoping Review (2017-2021) references and PRISMA diagram. [file Presentation_3.pdf]

Supplementary Material 3.
Scoping Review (2017-2021) references and PRISMA diagram

Following evaluation of the KS transition passport, we conducted an updated literature search (January 2017 - September 2021) in PubMed using the key word "Klinefelter syndrome". The PRISMA diagram and list of identified relevant articles are provided below.

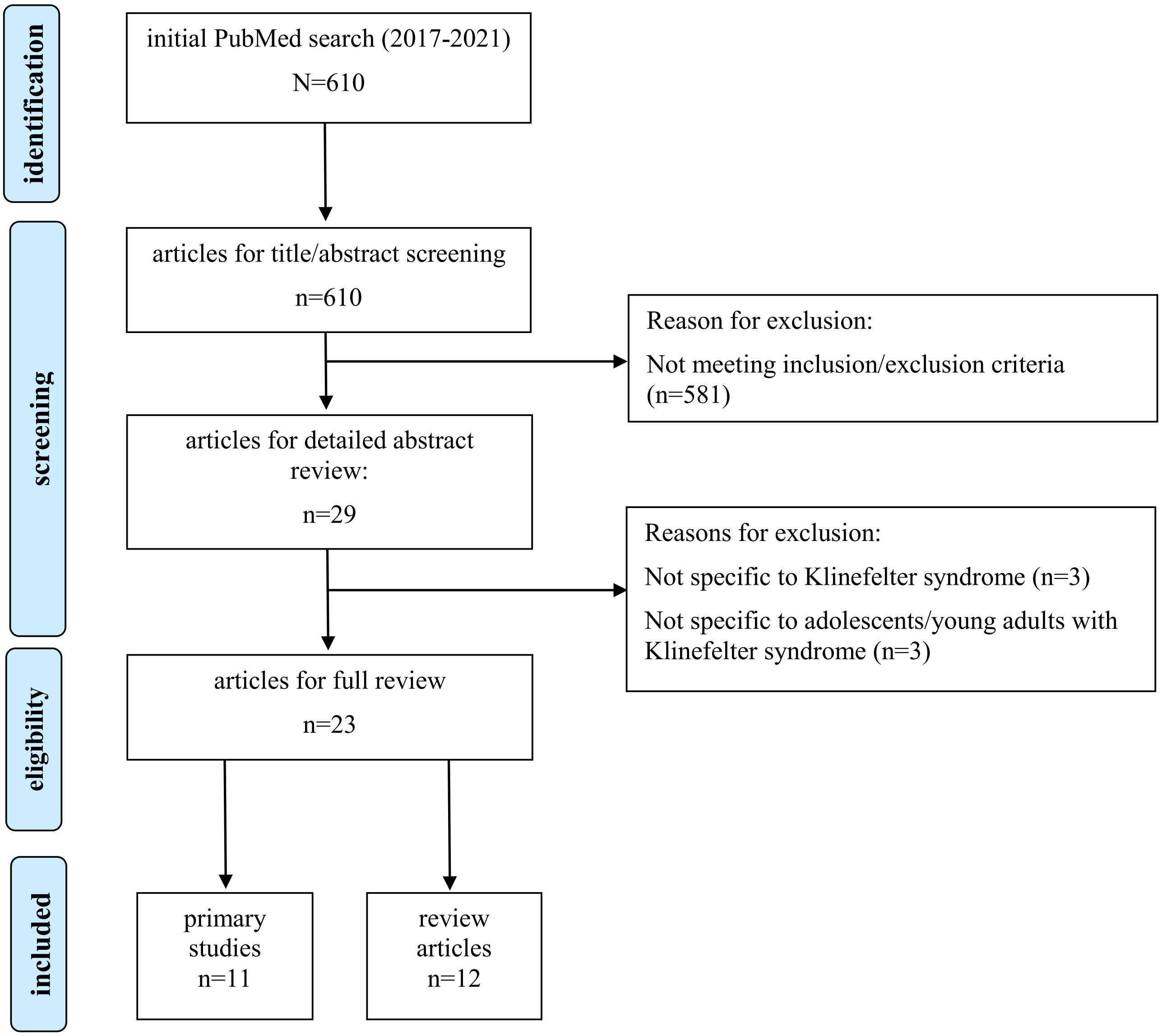

Identified relevant articles (n=23)

1. Akcan N, Poyrazoglu S, Bas F, Bundak R, Darendeliler F. Klinefelter Syndrome in Childhood: Variability in Clinical and Molecular Findings. J Clin Res Pediatr Endocrinol. 2018; 10(2):100-107. doi:10.4274/jcrpe.5121

2. Flannigan R, Patel P, Paduch DA. Klinefelter Syndrome. The Effects of Early Androgen Therapy on Competence and Behavioral Phenotype. Sex Med Rev. 2018; 6(4):595-606. doi:10.1016/j.sxmr.2018.02.008

3. Gravholt CH, Chang S, Wallentin M, Fedder J, Moore P, Skakkebaek A. Klinefelter Syndrome: Integrating Genetics, Neuropsychology, and Endocrinology. Endocr Rev. 2018; 39(4):389-423. doi:10.1210/er.2017-00212

4. Skakkebaek A, Moore PJ, Chang S, Fedder J, Gravholt CH. Quality of life in men with Klinefelter syndrome: the impact of genotype, health, socioeconomics, and sexual function. Genet Med. 2018; 20(2):214-222. doi:10.1038/gim.2017.110

5. van Rijn S, de Sonneville L, Swaab H. The nature of social cognitive deficits in children and adults with Klinefelter syndrome (47,XXY). Genes Brain Behav. 2018; 17(6):e12465. doi:10.1111/gbb.12465

6. Van Saen D, Vloeberghs V, Gies I, et al. When does germ cell loss and fibrosis occur in patients with Klinefelter syndrome? Hum Reprod. 2018; 33(6):1009-1022. doi:10.1093/humrep/dey094

7. Williams LA, Pankratz N, Lane J, et al. Klinefelter syndrome in males with germ cell tumors: A report from the Children's Oncology Group. Cancer. 2018; 124(19):3900-3908. doi:10.1002/cncr.31667

8. Bearelly P, Oates R. Recent advances in managing and understanding Klinefelter syndrome. F1000Res. 2019; 8. doi:10.12688/f1000research.16747.1

9. Chan YM, Feld A, Jonsdottir-Lewis E. Effects of the Timing of Sex-Steroid Exposure in Adolescence on Adult Health Outcomes. J Clin Endocrinol Metab. 2019; 104(10):4578-4586. doi:10.1210/jc.2019-00569

10. Hanna ES, Cheetham T, Fearon K, et al. The Lived Experience of Klinefelter Syndrome: A Narrative Review of the Literature. Front Endocrinol (Lausanne). 2019; 10:825. doi:10.3389/fendo.2019.00825

11. Zganjar A, Nangia A, Sokol R, Ryabets A, Samplaski MK. Fertility in Adolescents With Klinefelter Syndrome: A Survey of Current Clinical Practice. J Clin Endocrinol Metab. 2020; 105(4). doi:10.1210/clinem/dgz044

12. Chang S, Christiansen CF, Bojesen A, Juul S, Munster AB, Gravholt CH. Klinefelter syndrome and testosterone treatment: a national cohort study on thrombosis risk. Endocr Connect. 2020; 9(1):34-43. doi:10.1530/EC-19-0433

13. Chen W, Bai MZ, Yang Y, et al. ART strategies in Klinefelter syndrome. J Assist Reprod Genet. 2020; 37(9):2053-2079. doi:10.1007/s10815-020-01818-2

14. Davis SM, DeKlotz S, Nadeau KJ, Kelsey MM, Zeitler PS, Tartaglia NR. High prevalence of cardiometabolic risk features in adolescents with 47,XXY/Klinefelter syndrome. Am J Med Genet C Semin Med Genet. 2020; 184(2):327-333. doi:10.1002/ajmg.c.31784

15. Kyritsi EM, Kanaka-Gantenbein C. Autoimmune Thyroid Disease in Specific Genetic Syndromes in Childhood and Adolescence. Front Endocrinol (Lausanne). 2020; 11:543. doi:10.3389/fendo.2020.00543

16. Mason KA, Schoelwer MJ, Rogol AD. Androgens During Infancy, Childhood, and Adolescence: Physiology and Use in Clinical Practice. Endocr Rev. 2020; 41(3)doi:10.1210/endrev/bnaa003

17. Masterson TA, 3rd, Nassau DE, Ramasamy R. A clinical algorithm for management of fertility in adolescents with the Klinefelter syndrome. Curr Opin Urol. 2020; 30(3):324-327. doi:10.1097/MOU.0000000000000757

18. Rogol AD. Human sex chromosome aneuploidies: The hypothalamic-pituitary-gonadal axis. Am J Med Genet C Semin Med Genet. 2020; 184(2):313-319. doi:10.1002/ajmg.c.31782

19. Zitzmann M, Rohayem J. Gonadal dysfunction and beyond: Clinical challenges in children, adolescents, and adults with 47,XXY Klinefelter syndrome. Am J Med Genet C Semin Med Genet. 2020; 184(2):302-312. doi:10.1002/ajmg.c.31786

20. Butler G. Incidence of gynaecomastia in Klinefelter syndrome adolescents and outcome of testosterone treatment. Eur J Pediatr. 2021; 180(10):3201-3207. doi:10.1007/s00431-021-04083-2

21. Fabrazzo M, Accardo G, Abbondandolo I, et al. Quality of life in Klinefelter patients on testosterone replacement therapy compared to healthy controls: an observational study on the impact of psychological distress, personality traits, and coping strategies. J Endocrinol Invest. 2021; 44(5):1053-1063. doi:10.1007/s40618-020-01400-8

22. Vogiatzi MG, Davis SM, Ross JL. Cortical Bone Mass is Low in Boys with Klinefelter Syndrome and Improves with Oxandrolone. J Endocr Soc.2021; 5(4):bvab016. doi:10.1210/jendso/bvab016

23. Zitzmann M, Aksglaede L, Corona G, et al. European academy of andrology guidelines on Klinefelter Syndrome Endorsing Organization: European Society of Endocrinology. Andrology. 2021; 9(1):145-167. doi:10.1111/andr.12909
