# Supplementary material for: Navigating Disrupted Puberty: Development and Evaluation of a Mobile-Health Transition Passport for Klinefelter Syndrome
Source: Front Endocrinol (Lausanne). 2022 Jun 24;13:909830. doi: 10.3389/fendo.2022.909830 (PMC9264386; doi:10.3389/fendo.2022.909830)
Supplement: Supplemental Material 4 — Passport instructions. [file Presentation_4.pdf]

A guide for the patient with Klinefelter Syndrome (KS)

This guide will help you:

- Understand Klinefelter syndrome (KS).
- Know what to expect about KS.
- Organize your results to share with doctors and nurses.

How do I use this guide?

- Click on the icons to skip to the section you want to see
- A short description for each section is shown below

The syndrome in short

- This section explains why some men have KS.
- It describes symptoms of KS.
- It explains how KS is diagnosed.

At birth

Childhood

Puberty

Young adult

Adult

These sections explain how KS might affect your health and wellness at different times of your life.

A guide for the patient with Klinefelter Syndrome (KS)

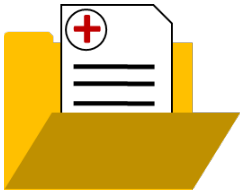

The sections below are to help you:

- Understand treatment for KS.
- Know what you can do to stay healthy.
- Organize your medical information and results.
- Save your information to share with health care providers.

Treatment

- This section explains treatment to help to say healthy
- It explains the importance of hormones

Fertility

- This section explains how KS affects fertility
- It also explains special fertility treatment

My follow-up

- This section explains how often you need certain to help you stay healthy.

My parameters

- This section lets you record important information about your medical history.

My exams

- This section lets you record your test results.

My treatment

- This section lets you record the types of medication and treatments you have had.

My contacts

- This section lets you record the contact information for your health care providers.
