# Supplementary material for: Navigating Disrupted Puberty: Development and Evaluation of a Mobile-Health Transition Passport for Klinefelter Syndrome
Source: Front Endocrinol (Lausanne). 2022 Jun 24;13:909830. doi: 10.3389/fendo.2022.909830 (PMC9264386; doi:10.3389/fendo.2022.909830)
Supplement: Supplemental Material 5 — Klinefelter syndrome transition passport. [file Presentation_5.pdf]

# Klinefelter syndrome

A guide for the patient
with Klinefelter Syndrome

canton de vaud
LIBERTÉ ET PATRIE


The syndrome in brief

Birth and Infancy

My follow up

Childhood

My contacts

Puberty

Recommended tests

Young adult

My results

Adult

My treatment

# The syndrome in brief

## The syndrome in brief

Klinefelter syndrome (KS) is the set of signs or symptoms of men born with at least one extra X chromosome.

## Chromosomes

Human cells usually contain 46 chromosomes (23 pairs), including 2 «sex chromosomes» (X and Y). We call them "sex chromosomes" because normally women have two X-chromosomes (46, XX) and men have one X and one Y chromosome (46, XY). Sometimes there is a problem with the number of chromosomes. There can be less than 46 or more than 46.

## 47, XXY

One possible problem is when a male has more than one X chromosome. In men with KS, 47, XXY is the most common chromosome number variation.

## Consequences

Chromosomes contain genes. An extra X chromosome can cause genes to act (be expressed) differently. An extra X chromosome mainly affects the testes, which as a result do not develop and function normally. An extra X chromosome can also impact other body functions such as metabolism (how your body changes food to energy).

## Frequency

Having an extra chromosome is not very uncommon. About 1 in 500-600 males have KS (47, XXY). KS is not passed on to children.

## Signs and symptoms

The signs and symptoms of KS vary:

- tall stature with long arms/legs
- puberty that usually starts normally, but may be incomplete
- small testes
- low hormone (testosterone) levels
- difficulty to have children without medical therapy

Some boys and men have only subtle signs:

- learning difficulties (especially language) or attention problems
- feeling tired (fatigue)
- moody (irritability)
- anxiety
- lack of self-confidence

In most cases, KS is diagnosed during puberty or adulthood. We think that most men with KS (2 out of 3) are never diagnosed.

## Diagnosis

KS diagnosis is made by testing the chromosomes (karyotype) and measuring hormone levels in the blood. A chromosome test can be done before birth or at any time during life.

## Insurance

The costs for care and treatment are usually covered by health insurance. Depending on where you live, some special requests and forms must be completed.

# Birth and Infancy

## At birth,

At birth, a male with Klinefelter syndrome (KS) typically has normal weight, height, sleep, eating and behavior. Sometimes, one or both testes (testicles) are not descended in the scrotum (cryptorchidism) and the penis is unusually small (micropenis).

## Karyotype

When the testes or penis are not normal, a test of chromosomes (karyotype) can be done to make a KS diagnosis.

## Hormones

At 2-3 months of age, other tests can be done:

- hormones from the testes: testosterone , AMH (antimüllerian hormone) and Inhibin B
- hormones from the brain (pituitary gland) that control the testes: LH (luteinizing hormone) and FSH (follicle stimulating hormone)

## Treatment

Testes that are not located in the scrotum can be fixed by surgery. It is best if surgery is done by the age of 2 years. If the penis is unusually small, testosterone can be given to increase the size.

# Childhood

Many children with Klinefelter syndrome (KS) have normal intelligence and grow and develop normally.

## Some children may have:

- delayed speech and language
- learning difficulty or problems understanding (comprehension)
- reading learning difficulties
- attention deficit disorder
- social problems (feel shy, reserved or insecure)
- problems with coordination

If these problems occur, speech therapy and extra school support can be very helpful.

## Other signs can be:

- unusually long arms and legs.
- bones that are slow to mature (delayed bone maturation)
- curved spine (scoliosis)
- dental problems (the molars)

[Treatment in Children and Teenagers]

# Puberty

Puberty starts when the pituitary gland (in the brain) starts making hormones (LH and FSH) that stimulate the testes. The testes will grow and start to make hormones (testosterone) and sperm (spermatozoa). However, in males with Klinefelter syndrome (KS) the testes do not develop normally.

## Males with KS may have:

- firm testes (about the size of a hazelnut in its shell) that stay small (4-6 ml)
- low male hormones (testosterone)
- low sperm and fertility problems (infertility) in adulthood

## Sometimes other signs occur. These may include:

- late (delayed) or incomplete puberty
- low muscle mass
- increased fat mass
- little facial and body hair
- low bone mass (osteoporosis)
- unusual body proportions (tall height, large arm span)
- gynecomastia (breast development)
- reading learning difficulties
- problems with body image or low self-esteem

[Treatment in Children and Teenagers]

# Young adult

Young adulthood (16 to 25 years-old) is a time when many changes occur:

- increased independence
- increased role of peers
- sense of one's body (body image)
- sense of one's identity
- developing sexuality

This period from 16-25 years is often called "transition". It describes the change from childhood to adulthood.

Young adults with Klinefelter syndrome (KS) benefit from care by different types of providers (a multidisciplinary team) including:

- family physician (primary care)
- pediatric / adult endocrinologists
- fertility specialist (urologist)
- speech therapist
- geneticist
- behavioral health specialist / psychologist / psychiatrist
- support groups

[Treatment in Children and Teenagers]

# Adult

## Adult

With proper treatment, men with Klinefelter syndrome (KS) can lead a pretty normal life. Regular medical appointments are important because men with KS have increased risk for certain health problems including:

- feeling frustrated or distressed (e.g., anxiety, depression)
- excess fat around the belly. Being overweight or obese increases the risk of other health problems of the blood vessels (e.g., atherosclerosis) and how your body changes food to energy (e.g., type 2 diabetes)
- weak, fragile bones (osteoporosis)
- dental problems (taurodontism)
- curved spine (scoliosis)
- blood clots in the legs (thrombosis)
- autoimmune diseases (e.g., lupus, rheumatoid arthritis, thyroid disorders)
- Rarely: increased cancer risk (e.g., breast, blood, bone marrow or lymph nodes).

Fertility problems are covered in a different section.

Treatment in Adults

# Treatment in Children and teenagers

A balanced diet and regular physical activity are recommended for all males with Klinefelter syndrome (KS). Regular medical and dental visits are also important. Most children and teens do not need a specific therapy. Instead, care should be tailored to individual needs and may include:

- additional support at school (e.g., special education, tutors)
- discussing concerns about body-image or self-esteem with caregivers or a behavioral health specialist.

Most teens with KS will start puberty normally.

Some boys will need testosterone replacement therapy (TRT). TRT is used to help develop muscles, signs of puberty (e.g., facial hair, deepening of voice) and sexual function (erections), but will not make the testes grow in size. TRT is also very important for bone health and may increase energy and indurance. Regular doctor visits are important to monitor TRT.

# Treatment in adults

## Hormonal treatment in adults

Most adult men with KS have low testosterone levels. Testosterone replacement therapy (TRT) is recommended to increase testosterone to normal levels. TRT is also very important for bone health. There are several types of TRT. Common types of TRT include injections (given by a healthcare provider or the patient) or applying a gel to the skin (daily). You can talk to your doctor about options for TRT.

TRT helps improve sexual desire (libido) and erections. TRT does not increase the size of the testes. TRT does not help improve fertility (sperm).

## Breast tissue (gynecomastia)

In some cases, breast tissue (gynecomastia) is very bothersome. Specialized surgeons can perform surgery to reduce the size of the breast - but it is rarely necessary.

## Metabolism

Metabolism refers to how your body changes food to energy. Men with KS have increased risk for metabolic problems including being overweight or obese. A healthy balanced diet and regular physical exercise is recommended for all men with KS. This is important to control weight gain and have a healthy metabolism. Low testosterone can cause metabolic problems so it is important to take TRT as prescribed by your doctor. Stopping TRT can increase your risk for metabolic problems like obesity and type 2 diabetes.

## Other treatments

Some adults with Klinefelter syndrome and certain associated health problems will need to adress these with specific treatments.

Fertility disorders are covered in a different section.

# Fertility

## Fertility

During normal puberty, special cells in the testes develop to make spermatozoa (sperm). In teens with KS, the special cells do not develop and start to die off during the teen years. The loss of cells causes infertility. Sperm is rarely seen in the semen of men with KS (low or no sperm).

Testosterone replacement therapy (TRT) will not improve fertility. Men with KS need special techniques and surgery to get sperm (e.g., testicular sperm extraction, TESE). TESE can get sperm in about half (i.e., 30-50%) of men with KS.

Sperm found during surgery can be frozen and stored (cryopreservation). The sperm can be used by fertility specialists for assisted fertility (in vitro fertilization, IVF). IVF is a procedure done in a laboratory. Sperm is injected into an egg to fertilize it. The cells are grown in the laboratory for a short time and then implanted into the woman's uterus.

Some patients are able to have children using assisted fertility, but these techniques are not always successful.

# Recommended tests

Adapted from Gravholt et al. Endocrine Reviews, 2018

| Test | Childhood | Puberty | Adult |
|---|---|---|---|
| Growth | 1x/yr | 1-2x/yr | |
| Weight and body composition | 1x/yr | 1x/yr | 1x/yr |
| Testosterone | | 2x/yr | 1x/yr |
| LH, FSH | | 2x/yr | 1x/yr |
| Estradiol, AMH, Inhibin B | | 1x/yr | 1x/yr |
| Calcium, Phosphate, alkaline phosphatase, Vitamine D | | 1x/yr | 1x/yr |
| Blood count (hemoglobin, hematocrit) | | 1x/yr | 1x/yr |
| Liver enzymes | | 1x/yr | 1x/yr |
| Lipid profile | | 1x/yr | 1x/yr |
| Fasting glucose, Insulin, HbA1c | | 1x/yr | 1x/yr |
| PSA | | | 1x/yr |
| Bone density (DXA) | | 1x/2-3 yr | 1x/2-3 yr |
| Auto-immune screening (e.g. thyroiditis, celiac disease) | X | X | X |
| Cardiovascular assessment | | | X |
| Atheroscelorisis risk evaluation | | | X |
| Tumor screening | | | X |
| Testosterone treatment (if high LH) | | X | X |
| Fertility consultation | | X | X |
| Sperm count | | X | X |
| Testicular biopsy / TESE | | | X |
| Neuropsychologist (if needed) | X | X | X |
| Academic support (if needed) | X | X | X |
| Physical activity & balanced diet | X | X | X |

# My follow-up

## Karyotype

| | |
|---|---|
| Test date | |
| Laboratory | |

## Birth

| | | | |
|---|---|---|---|
| Weight | | Length | |
| Cryptorchidism | | Penile size | |

## Minipuberty (0-6months)

| | | Date | |
|---|---|---|---|
| LH | | FSH | |
| Testosterone | | Inhibin B | |
| AMH | | Other | |

## Infancy / Childhood

| | |
|---|---|
| Increased height | |
| Delayed growth/milestones | |
| Dental problems | |
| Motor difficulties | |
| Delayed speech | |
| Learning difficulties | |
| Attentional problems | |
| Psychologist | |
| Other | |

## Puberty

| | |
|---|---|
| First signs of puberty | |
| First signs of growth spurt | |
| Other | |

## Other health problems

| | | Date of diagnosis | |
|---|---|---|---|
| Gynecomastia | | Date | |
| Obesity | | Date | |
| Diabetes | | Date | |
| Osteoporosis | | Date | |
| Orthopedic problems | | Date | |
| Phlebitis | | Date | |
| Autoimmune diagnosis | | Date | |
| Other | | Date | |

## Fertility consultation

| | | Date of diagnosis | |
|---|---|---|---|
| Sperm count/analysis | | Date | |
| Cryopreservation | | Date | |
| Microdissection | | Date | |

# My treatments

| Treatment | Dose | Frequency | Start-Date | End-Date |
| --- | --- | --- | --- | --- |
| | | | | |
| | | | | |
| | | | | |
| | | | | |

# My contacts

| Contacts | Name | City | Telephone |
|---|---|---|---|
| Pediatrician | | | |
| Pediatric endocrinologist | | | |
| Speech & Language | | | |
| Psychologist | | | |
| Fertility specialist | | | |
| Primary care provider | | | |
| Adult endocrinologist | | | |

| Links | |
|---|---|
| Association for X and Y Chromosome Variations | National Institute of Health |
| Klinefelter's Syndrome Association - UK | Klinefelter Syndrome Association - Canada |
| Deutsche Klinefelter-Syndrom Vereinigung e.V. | AXYS Australia |
| Verein Klinefelter-Syndrom Schweiz | Association de Porteurs d'Anomalies Chromosomiques |

# My Results

| Growth | Value | Units | Date |
|---|---|---|---|
| Height | | | |
| Weight | | | |
| BMI | | | |
| Armspan | | | |

| Testicular function | Value | Units | Date |
|---|---|---|---|
| Testosterone | | | |
| Estradiol E2 | | | |
| LH | | | |
| FSH | | | |
| AMH/MIS | | | |
| Inhibin B | | | |
| Testicular ultrasound | | | |
| Sperm count | | | |
| Biopsy/TESE | | | |
| Other | | | |

| Bone health | Value | Units | Date |
|---|---|---|---|
| Vitamine D(25 OH)) | | | |
| Calcium | | | |
| Phosphate | | | |
| Alk. Phos. | | | |
| Bone density (DXA) | | | |
| Other | | | |

| Metabolism | Value | Units | Date |
|---|---|---|---|
| HbA1c | | | |
| Glucose | | | |
| Insulin | | | |
| Total Cholesterol | | | |
| HDL cholesterol | | | |
| Other | | | |
| Other | | | |

| Other | Value | Units | Date |
|---|---|---|---|
| TSH | | | |
| IGF1 | | | |
| Hematocrit | | | |
| Liver enzymes) | | | |
| Thyroid | | | |
| Celiac | | | |
| Other | | | |
| Other | | | |
